# The Telomeric Complex and Metabolic Disease

**DOI:** 10.3390/genes8070176

**Published:** 2017-07-07

**Authors:** Henriette Kirchner, Fozia Shaheen, Hannes Kalscheuer, Sebastian M. Schmid, Henrik Oster, Hendrik Lehnert

**Affiliations:** 1 First Department of Medicine, University of Lübeck, Ratzeburger Allee 160, 23562 Lübeck, Germany; henriette.kirchner@uksh.de (H.K.); hannes.kalscheuer@uksh.de (H.K.); sebastian.schmid@uksh.de (S.M.S.); 2 Stratlab Ltd., March, Cambridgeshire PE15 8QD, UK; Fozia_Shaheen@hotmail.co.uk; 3 German Center for Diabetes Research (DZD), Ingolstädter Landstraße 1, 85764 Neuherberg, Germany; 4 Institute of Neurobiology, University of Lübeck, Ratzeburger Allee 160, 23562 Lübeck, Germany; henrik.oster@uni-luebeck.de

**Keywords:** metabolic diseases, leucocyte telomere length, diabetes, obesity telomere shortening

## Abstract

The attrition of telomeres is believed to be a key event not only in mammalian aging, but also in disturbed nutrient sensing, which could lead to numerous metabolic dysfunctions. The current debate focuses mainly on the question whether telomere shortening, e.g., as a heritable trait, may act as a cause or rather represents a consequence of such chronic diseases. This review discusses the damaging events that ultimately may lead or contribute to telomere shortening and can be associated with metabolic diseases.

## 1. Introductory Remarks

The attrition of telomeres is a hallmark of mammalian aging, but may also play a role in the disturbance of nutrient sensing, leading to numerous metabolic dysfunctions. A large body of evidence suggests shorter telomeres are a risk factor for age-related pathologies such as insulin resistance, overt diabetes mellitus, cardiovascular disease or neurological disorders such as Alzheimer’s disease. The current debate focuses mainly on the problem whether telomere shortening, e.g., as a heritable trait, represents the cause of such chronic diseases or whether it rather reflects a disease consequence. Either way, loss of chromosomal DNA end-protection will itself lead to a deterioration of numerous cellular functions. In this article we will, against the background of metabolic diseases, discuss several damage-causing events that ultimately lead or contribute to telomere shortening. We will also recapitulate recent literature on the a priori independence of metabolic diseases and telomere length, thus advocating the causal role in this attrition for diseases such as pre-diabetes and overt diabetes. Finally, the emerging extranuclear role of telomerase and, in particular, its catalytic subunit, telomerase reverse transcriptase (TERT), in the regulation of metabolic functions, will be presented and discussed as a possible target for the treatment of metabolic disorders.

## 2. Principles of Telomere Biology

Telomeres themselves do not encode proteins, but are essential because of their multiple biological functions. These include maintenance of chromosomal structure and stability, the prevention of chromosome end-to-end fusion and eventually the determination of the life span of cells. Telomere length is thus considered the life-clock of a cell. The homeostasis of telomere length is essential for the survival of cells and the whole organism. Telomere shortening induces DNA damage, cellular senescence and apoptosis, and triggers associated ageing disorders. Conversely, maintenance or elongation of telomere length is linked with cell immortality and, eventually, tumorous growth [1]. In eukaryotes, chromosomes are linear with the critical consequence that chromosomal ends might be recognized as DNA strand breaks by the cellular DNA repair system. Erroneous repair could therefore lead to chromosomal end-to-end fusion, genomic instability and apoptosis [2]. Together with the shelterin and CST (CTC1, STN1, and TEN1) complexes, telomeres prevent such unwanted DNA damage responses, thus protecting the genomic information at the end of chromosomes. Telomeres themselves consist of 2 to 20 kb of double-stranded DNA (TTAGGG) repeats ending in a short single-stranded G-rich overhang. This double- and single-stranded telomeric DNA is associated with the shelterin complex that consists of the six telomeric-DNA-binding proteins: telomere repeat factor 1 (TRF1), telomere repeat factor 2 (TRF2), repressor/activator protein 1 (RAP1), TRF1-interacting nuclear factor 2 (TIN2), protection of telomeres 1 (POT1), and adrenocortical dysplasia homolog (ACD) [3]. Shelterin harbors chromatin remodeling activity and shelterin-mediated compaction of telomeric chromatin protects telomeres against DNA damage responses [4,5] as shelterin proteins recruit the telomerase complex to the chromosomal ends by an interaction with TERT [6,7]. CST is a multiprotein complex in higher eukaryotes consisting of CTC1, STN1 and TEN1 and localizing specifically to the single-stranded overhang of telomeres. [8]. It is most important for chromosome end capping and regulation of telomere length [9,10,11]. Moreover, downregulation of the CST complex leads to excessive telomerase activity and consequently telomere elongation. Thus, the CST complex is crucial for regulating telomerase action [11].

From a pathophysiological perspective, telomere shortening indicates the cumulative memory of genomic damage and the cell’s exposure to oxidative stress, although the role of the latter in telomere attrition is still poorly understood [12]. Telomere length regulation is an extremely complex trait shaped by numerous factors including genetic, epigenetic, environmental, and further unknown events [13,14,15]. It is altered by somatic stress from, e.g., malnutrition, chronic inflammation or low physical activity, and nonsomatic stressful life factors such as psychological burden or low socioeconomic status. Understood this way, telomere length can be regarded as both a disease potentiator and mortality predictor [16].

In addition to these mechanisms, a large and still growing body of evidence points towards a major role of heritability of telomere length with a reported variance between 36% and 90% [17,18,19]. This adds another important aspect to the still biological conundrum of the regulation of telomere morphology and function, namely the role of telomeres to mechanistically impact on cellular function and ultimately the development of disease; independent from its role harmfully responding to internal and external signals. This aspect is of major importance in metabolic and neurological disease, bears a high relevance for longitudinal studies and will be alluded to in more detail below.

In understanding the association of telomeres with disease states, knowledge of the cellular mechanisms affecting telomere stability is crucial. Telomere maintenance depends on the activity of the enzyme telomerase together with shelterin and the CST complex. Telomerase is a ribonucleoprotein complex that consists of the catalytic subunit TERT, and the non-coding telomerase RNA component (TERC) which serves as a template for telomere sequence elongation by TERT [20]. A third subunit of the telomerase complex is telomerase-associated protein 1 (TEP1), or dyskerin. TEP1 and TERC are constitutively expressed, rendering the enzymatic activity of telomerase dependent on the transcription of TERT. TERT expression is epigenetically controlled through mechanisms such as DNA and histone methylation, or histone acetylation [21]. Beyond its role in the telomerase complex, TERT possesses numerous extranuclear functions critical for mitochondrial and metabolic homeostasis (see below).

Finally, in gene-encoding proteins that are required for intact telomere structure or repair mechanisms of telomeres may lead to critically short telomeres giving rise to complex telomere biology disorders (TBDs), which can be highly debilitating and life-threatening [22]. Increased expression of TERT is associated with certain tumors [23] and telomerase activation through abrogation of transcriptional silencing of TERT can lead to the development of high-risk cancers such as neuroblastoma [24]. Deciphering the complex telomeric biology is crucial for understanding associated diseases and, vice versa, the molecular phenotyping of telomere diseases will help to further clarify telomere function.

## 3. Diet, Nutrition and Telomeric Function

Lifestyle and dietary patterns have in several studies been investigated for their impact on telomeric complex components and telomere length. Based on findings that specific dietary components such as whole grains, nuts, vegetables and fruits [25,26,27,28,29], through their anti-inflammatory and antioxidative properties, may positively affect overall mortality, it has been speculated that this may be achieved by reducing telomere shortening. In particular, it was speculated that a dietary pattern characterized by the intake of whole grains, nuts, vegetables and fruits, which has been shown to possibly favor longevity, might be associated with increased telomere length. In a first attempt to study the association between certain macronutrients and telomere length, habitual intake of numerous food items including whole grains, fruits, vegetables, fish, coffee, fried food, red and processed meat or sugar-sweetened soda was correlated against leukocyte telomere length (LTL) [28]. Surprisingly, and after adjustment for several demographic and dietary aspects, only the intake of processed meat was found to be associated with telomere length in an inverse manner [28]. The other components did not reveal any association. However, this study is limited by a relatively small sample size of 840 participants and employing an exploratory approach rather than testing for multiple hypotheses. In a much larger, though still cross-sectional study, the association between LTL, diet and other lifestyle factors was studied in a cohort of 2284 female participants from the Nurses’ Health Study [17]. In this trial, waist circumference and polyunsaturated fatty acid intake—specifically linoleic acid—were inversely correlated with LTL, while a diet high in cereal fibre showed a positive association. These data are in accordance with another pilot trial, in which the adoption of a healthier lifestyle (including dietary change towards a low-fat, plant-based diet, aerobic exercise, stress management and supplementation with various minerals and vitamins) led to an increase in telomerase activity in peripheral blood mononuclear cells [29]. Based on these findings, it is tempting to speculate that dietary components such as cereal fibre and whole grains may favorably affect telomere length through anti-inflammatory and antioxidative mechanisms. A similar mechanism may account for the positive effects of marine omega-3 fatty acids, that have been shown to reduce telomere shortening over a median of six years in a prospective trial in male patients with coronary artery disease [30]. Interestingly, dietary supplementation with omega-3 fatty acids is associated with lower concentrations of F2 isoprostanes, an established surrogate marker for systemic oxidative stress, and with higher levels of the antioxidant enzymes catalase and superoxide dismutase [31]. The Mediterranean diet contains most of the above mentioned beneficial foods as it is rich in fruits and vegetables, grains, legumes and sea fish. Therefore, the Mediterranean diet is claimed to be one of the healthiest nutritional regimes at all being associated with low mortality and morbidity for several chronic diseases [32,33]. A possible mechanism is that adherence to the Mediterranean diet is associated with longer LTL, independently of factors that are known to influence telomere attrition [34]. An increase of telomerase activity after meal consumption seems to play a particularly important role [34]. Thus, a mixture of healthy foods that have been shown to harbor individual effects on LTL, such as the Mediterranean diet, as opposed to ingestion of single food items with effects on LTL, might be the best option to extend health and lifespan [35]. Nevertheless, in the future it will be important to study lasting protective effects in larger trials.

From a different angle, there is a steadily increasing interest in so-called *epigenetic* diets and, in this context, on the effect of bioactive dietary components on telomeric activity through epigenetic modification of the TERT promoter. This interest is mainly based on the assumption that specific compounds may exert anti-cancer effects by inhibiting telomerase activity. A detailed description is beyond the scope of this article, but it should be noted that particular food items such as tea (through polyphenols), soybeans, grapes (resveratrol), or turmeric (curcumin) may either inhibit telomerase activity by demethylating the TERT promoter (tea polyphenols), to stimulate expression of TERT mRNA (soybeans, curcumin) or to increase TERT activity through suppression of sirtuin-1 activity (resveratrol) [21]. To which extent these effects are associated with metabolic changes, is currently unknown.

## 4. Impact of—and on—Obesity

The relationship between obesity and telomere morphology is still unresolved and puzzling. A recent systematic review on this topic concluded that a negative correlation between obesity and telomere length is likely, but with only weak to moderate strength and a large amount of heterogeneity [36]. One of the first trials studying this interaction analyzed data from the Malmö Diet and Cancer Cohort (MDCC) and the Northern Sweden MONICA project [37]. Significant or borderline associations with telomere length were found for several phenotypical obesity parameters such as body mass index (BMI), weight and waist circumference in women. This was not shown for men, in which, on the other hand, a positive borderline association between High-density lipoproteins (HDL)-cholesterol levels and telomere length and a negative one for 2 h glucose increase following an oral glucose load was shown. Similarly, in the Nurses’ Health Study, which was already alluded to above, an adverse body composition, such as an increased waist-to-hip-ratio, was inversely correlated to LTL [17], supporting the notion that this association might herald and mediate chronic disease risk.

A French trial on obese boys and girls aged 2–17 years revealed significantly shorter leukocyte telomeres in obese children versus their non-obese counterparts [38]. While this study did not put forward any mechanistic explanation, it demonstrated a gender-independent relationship and rightly concluded that obese children are at an apparent higher biological age demanding early interventions to reduce the burden of future disease risk.

This inverse relation between obesity and telomere length is biologically plausible, since oxidative stress and the secretion of pro-inflammatory cytokines may be enhanced with increasing BMI. In comparison with genomic DNA, the guanine-rich telomeric sequence repeats clearly represent a rather unprotected target for oxidative damage [39]. Interestingly, in a very large study on 45,069 women and men from the Copenhagen General Population Study, telomere length not only decreased with seven base pairs per unit increase in BMI, it also decreased with nine base pairs for a doubling in C-reactive protein (CRP) levels, suggesting that inflammation plays a mechanistic role in this alteration of telomere length [40]. A damage of telomeric DNA through CRP-induced activation of the complement system may increase the generation of oxidative free radicals by neutrophils, thus affecting telomere stability [41].

To better understand the mechanisms of this interaction, prospective trials are clearly mandatory. As such, LTL and phenotypic markers of obesity were assessed in 2721 elderly subjects [42]. At baseline, LTL was significantly associated with several obesity features such as relative body fat and subcutaneous fat content, but not with BMI or visceral adiposity. At the seven-year follow-up, LTL was related to a positive change in BMI and percentage of body fat. Thus, these data suggest that telomere length may predict changes in body composition. Surprisingly, in this study, shorter LTL was associated with lower leptin levels. While the authors speculate that this action is plausible based on the role of leptin as an appetite-suppressant hormone and thus, higher leptin levels are associated with a healthier status, one has to keep in mind that leptin levels increase with body weight gain and lose their protective function through numerous mechanisms assembled under the term of leptin resistance. Accordingly, in another study, a significant negative association between relative telomere length and leptin levels was found [43]. Over the past decade or so, high leptin levels have clearly been linked to adverse metabolic and cardiovascular traits, contributing to inflammation, insulin resistance, high blood pressure or atherosclerosis [44,45]. Adiponectin, another adipokine with positive effects on glucose tolerance and cardiovascular traits, was positively—though in a borderline manner—associated with telomere length [43]. These findings suggest that adipokines may exert differential effects on telomere morphology—a research question that should be addressed in future studies. Similarly, this most likely bidirectional relationship between LTL and obesity or metabolic syndrome was addressed in another prospective study including over 1800 participants from the Netherlands Study of Depression and Anxiety [46]. Here, increased baseline waist circumference and blood glucose levels were associated with shorter LTL over the follow-up period. A greater increase in waist circumference at six years was, in addition, associated with larger telomere attrition. While still far from proposing a mechanistic link between telomere biology and the genesis of the metabolic syndrome, in particular visceral obesity, these data do support the hypothesis of a bidirectional link between these two entities. Recent and in this context highly interesting data show significantly shorter telomere lengths in subcutaneous as compared to visceral adipose depots—independent of body weight or type-2 diabetic state [47]. Again, this stresses the need to further explore the interaction of different adipokines and telomere length, given that certain adipokines such as leptin or nesfatin are preferentially expressed in subcutaneous versus visceral adipose tissue [48].

Underscoring the potential effect of body composition on telomere length, two prospective studies have shown that weight loss interventions such as placement of an intragastric balloon and bariatric surgery were associated with a reduced rate of telomere attrition. In a smaller trial conducted in 42 obese subjects, placement of a bioenteric intragastric balloon led to telomere lengthening most significantly in those patients who exhibited the shortest telomeres at baseline [49]. In a larger trial, telomere length was shown to increase following bariatric surgery in 142 patients [50]. The cohort from the Bruneck study served as a control, demonstrating that the post-surgery increase was different from telomere changes in an age- and sex-matched population. These findings lend further support to the idea that obesity and its metabolic and inflammatory sequelae may shorten telomere lengths and that this may be reversible by metabolic interventions.

## 5. Diabetes and Telomeres—A Reciprocal Liaison?

Numerous studies have shown that alterations of the telomere-telomerase system are associated with both type-1 (T1DM) and type-2 diabetes (T2DM). Shorter LTL is associated with T1DM [51] and T2DM, independent of race, gender and population (reviewed in [52]). Furthermore, the degree of oxidative stress, increased age and obesity correlates positively with telomere shortening in both T1DM and T2DM [53]. Accordingly, LTL in T2DM patients of a diverse ethnic background was found to be positively correlated with plasma total antioxidant status and carriers of a variation (the 866-A allele that is associated with lower *UCP2* transcription) in uncoupling protein 2 (UCP2) had shorter age-adjusted LTL than their homozygote counterparts [12]. The ubiquitously expressed UCP2 is considered a negative regulator of oxidative stress as it uncouples respiration from oxidative phosphorylation, thereby reducing production of reactive oxygen species (ROS) [54]. These findings were supported by another trial in a Han Chinese population [55]. The association of this *UCP2* mRNA decreasing promoter variant with reduced LTL suggests an impact of mitochondrial reactive oxygen species on LTL in T2DM. In addition to T1DM and T2DM, telomere shortening also has been shown to exist in women with gestational diabetes and their offspring [56] suggesting genetic or epigenetic inheritance or a transgenerational transmission of a pro-inflammatory state from the mother to the fetus. Most interestingly, telomere shortening has also been observed in the pancreatic islets of T2DM patients, affecting both beta- and, to a lesser extent, alpha-cells, thus possibly contributing to the impaired insulin secretion at later stages of the disease [52]. Reduced telomere length in alpha-cells, in turn, may contribute to a compromised secretion of glucagon in T2DM patients.

Numerous mechanisms may affect telomere attrition observed in the diabetic state. Chronic hyperglycemia, enhanced fatty acid secretion and nutritional overload can act in concert to increase oxidative stress, activate the protein kinase C (PKC) pathway, adversely interact with insulin signaling, or induce the secretion of pro-inflammatory cytokines. PKCs are a family of serine/threonine-related protein kinases that play key roles in many cellular functions. For instance, specific isoforms of PKC are involved in endothelial dysfunction, vascular permeability, angiogenesis, cell growth and apoptosis, vessel dilation, basement membrane thickening, and extracellular matrix expansion [57]. In addition, mitochondrial dysfunction might further enhance oxidative stress and inflammatory processes. Although not conclusive at present, telomere length appears to be associated with diabetic complications such as nephropathy or retinopathy. In male T2DM patients with proteinuria, LTL was significantly shorter than in T2DM patients without diabetic nephropathy [58] and in T1DM patients from the Finn Diane Study Group telomere shortening was predictive for the progression of nephropathy [59]. For the other relevant microvascular complication, diabetic retinopathy, there is at present only circumstantial evidence of telomere length changes. Since retinal pigment epithelial cells are highly susceptible to senescence and oxidative damage, both associated with T2DM, they may suffer from telomeric loss [60].

While there is clear evidence for an effect of the diabetic state and its metabolic perturbations on telomere integrity, this interaction may go both ways. In other words, short telomeres are not a mere phenomenon of the diabetic and pre-diabetic state, but may play an important role in the pathogenesis and disease progression of diabetes, particularly T2DM. Short telomeres possibly promote early beta-cell senescence, resulting in reduced cell mass and impaired insulin secretion. Also, as shown in mice, short telomeres contribute to metabolic disturbances through mitochondrial dysfunction [61]. Importantly, the deletion of TERC has been shown to lead to these sequelae [62]. In addition, short telomeres may impair insulin secretion by attenuating calcium-mediated insulin exocytosis [63]. Finally, and as a piece of indirect evidence, inhibiting activity of tumor suppressor p53 can reverse adipocyte cellular senescence and its associated insulin resistance [64].

This causal association of telomere length and T2DM development has been investigated in several well-designed studies. In a prospective population-based approach LTL and incident T2DM was studied [65]. Within a 15-year follow-up, 44 of 606 participants without T2DM at baseline developed diabetes with a hazard ratio for T2DM of 2.00 when comparing bottom and top quartiles of baseline LTL and of 2.31 when comparing the bottom and the remainder. The authors estimated an average risk of 31% to develop diabetes when pooling the data from three prospective trials [65]. In the Strong Heart Family Study, Zhao and colleagues [66] studied the association of LTL with the future risk of diabetes over a period of five years. Those within the lowest LTL quartile had an approximately twofold risk of developing T2DM, revealing a nonlinear relation between telomere length and diabetes risk independent from other risk factors. In a recent trial, monozygotic and dizygotic twins from the Danish Twin Registry were followed-up over a period over 12 years [67]. Main findings were that the shorter the LTL at baseline, the more pronounced was the increase in insulin resistance (an effect being additive to the BMI effect) and that insulin resistance did not contribute to telomere attrition over time. These very promising data unanimously suggest that altered telomere integrity has unfavorable effects on glucose metabolism and might play a major role in the pathogenesis of T2DM. Consequently, it will be of great importance to investigate the mechanisms mediating the compromising effect of short telomeres on insulin sensitivity.

In addition to the processes outlined above, studying the genetic regulation of telomeres and its impact on telomere length will be highly relevant. For instance, in a longitudinal cohort of healthy middle-aged females of Caucasian origin, the haplotypes of telomere maintenance genes in association with T2DM were explored [68]. An association between several specified haplotypes with genes coding for TRF1 and TEP1 with T2DM risk was found in this study. In genome-wide association studies, shortening of LTL and T2DM risk was found to be related to both genotypes of OBFC1 (oligonucleotide/oligosaccharide-binding folds containing 1) and telomerases such as TERC. On the contrary, a recent genome-wide association study could not find significant associations between genetically increased telomere length and risk for diabetes [69]. Clearly, there is a need to further explore the influence of genetic factors on both LTL and T2DM and their interaction with mitochondrial function (for review see [70]).

Acknowledging that telomere length might play a functional role in the pathogenesis of T2DM, antidiabetic medications affecting telomere length might not only shed light on this interaction, but also open pathways to novel diabetes treatments. As such, it has been shown that peroxisome proliferator-activated receptor gamma (PPAR-gamma) agonists as used in the treatment of T2DM and stimulating lipid uptake and glucose metabolism may simultaneously improve glucose tolerance, decrease oxidative stress and alleviate telomere attrition in myocardial cells from diabetic Otsuka Long-Evans Tokushima Fatty (OLETF) rats [71]. Additionally, the oral anti-diabetic dipeptidyl peptidase-4 (DPP-IV) inhibitor *sitagliptin* may prolong telomere length in patients with T2DM, as has been assumed for the incretin hormone glucagon-like peptide 1 (GLP-1) [52]. Although their role in glucose metabolism remains ambiguous, statins have in a cross-sectional study been shown to increase telomerase activity and telomere length [72]. Whether novel compounds currently applied in patients with telomere disease such as danazol [73] will improve glucose homeostasis and insulin sensitivity cannot be answered at present, but should be regarded as an interesting future perspective. Last but not least and probably more important for diabetes treatment, lifestyle changes may also improve not only insulin resistance, but also enhance telomerase activity and have—in a pilot study—been shown to maintain telomere stability in obese women [74].

## 6. Extranuclear Functions of TERT

It is becoming increasingly evident, that besides its important role in regulating and maintaining telomere length, TERT is exerting extranuclear functions beyond catalyzing the addition of repetitive tandem repeat sequences to telomeres. TERT has been identified to protect mitochondrial DNA from damage induced by oxidative stress [75]. Under conditions of oxidative stress nuclear export of TERT is induced with a subsequent increase of TERT in mitochondria, suggesting that TERT is transported to organelles where it is needed to ensure cell survival [76].

As discussed earlier, mitochondrial function is involved in intracellular fuel metabolism and TERT may regulate pathways controlling the utilization of glucose [77]. Downstream targets of insulin such as phosphoinositide 3-kinase (PI3K) interact with TERT function, i.e., inhibition of PI3K activity reduces TERT phosphorylation by Akt and its nuclear translocation [78]. In this way, insulin may affect the cytosolic functions of TERT. Our group has investigated basal and insulin-stimulated glucose uptake in different muscle cell lines with systematically altered TERT expression or activity. The data revealed a novel extranuclear function of TERT contributing to the regulation of basal glucose uptake that was—somewhat surprisingly—insulin insensitive [77]. Inhibition of TERT expression reduced glucose uptake in all cell types employed, while overexpression markedly increased the uptake. TERT was found to be constitutively associated with the glucose transporters (GLUT) 1, 4 and 12—again through an insulin insensitive interaction that did not require PI3K or mechanistic target of rapamycin (mTOR) pathways [77]. These data collectively show that TERT associates with GLUT proteins to regulate glucose transport.

Extratelomeric roles have also recently been described for components of the shelterin complex, which is central for the protection of telomeres, such as repressor/activator protein 1 (RAP1). In an important study, RAP1 knockout mice showed an early onset of obesity, increased abdominal fat mass, liver steatosis, and signs of glucose intolerance. Peroxisome proliferator-activated receptor alpha (PPAR-alpha), which regulates lipid and carbohydrate metabolism, and the major regulator of mitochondrial biogenesis, peroxisome proliferator-activated receptor gamma coactivator 1-alpha (PGC1-alpha), were identified as the most important factors negatively affected by RAP1 deletion [79].

These data render TERT and other components of the telomeric complex, e.g., RAP1, highly attractive targets for positively affecting glucose metabolism and phenotypic and biochemical sequelae of the metabolic syndrome. Enhancing TERT expression and activity might improve glucose uptake and intracellular utilization. Further studies are clearly needed to address this clinically important question. Intriguingly, it was found that voluntary running increases TERT in the myocardium and vessel wall in mice [80]. When employing pharmacological means or TERT overexpression, one has to bear in mind that there are two sides of the coin with TERT also being associated with anti-apoptotic and possibly tumorigenic effects. Novel approaches enhancing TERT expression in specifically targeted cells are mandatory and demanding at the same time.

## 7. Conclusions

**Figure 1:**
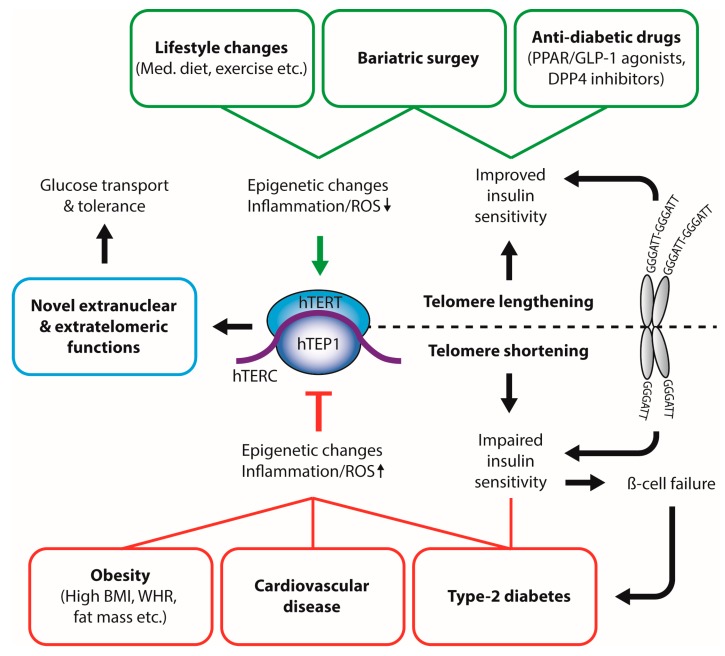
**Interplay between physiological and pathophysiological metabolic states and telomerase activity.** Dietary compounds, healthy lifestyle, and anti-diabetic therapies inhibit telomerase activity and lead to increased leukocyte telomere length (LTL). In contrast, obesity, increased fat mass, and type-2 diabetes result in decreased leukocyte telomere length, possibly through oxidative stress and inflammatory pathways. Telomere length can directly influence insulin sensitivity. BMI: body mass index; WHR: waist-to-hip ratio; Med. Diet: Mediterranean Diet; ROS: reactive oxygen species; hTERT: human telomerase reverse transcriptase; hTERC: non-coding telomerase RNA component; hTEP1: human telomerase-associated protein 1; PPAR: peroxisome proliferator-activated receptor; GLP-1: glucagon-like peptide 1; DPP4: Dipeptidyl peptidase-4.

In summary, this review outlines the important role of the telomeric complex as the proverbial canary in the coal mine on the one hand, reflecting the impact of various chronic diseases such as insulin resistance and diabetes through telomere attrition—an effect most likely due to mitochondrial dysfunction and a pro-inflammatory state. On the other hand, telomeres and their components appear to be causally related to the pathogenesis of metabolic and cardiovascular diseases, thus representing highly attractive therapeutic targets (Figure 1). The interaction of mitochondrial proteins, telomerase, TERT and shelterin components needs to be explored for their genetic basis and joint biochemical pathways in order to understand the mechanisms of disease and to eventually stratify the patient’s individual risk.
